# Molecular mechanism of Sishen pills in the treatment of diarrheal diabetic enteropathy based on network pharmacology

**DOI:** 10.1097/MD.0000000000030096

**Published:** 2022-09-02

**Authors:** Yunxia Tao, Chongchai Li, Tianshu Gao, Jingjing Huo

**Affiliations:** a Liaoning University of Traditional Chinese Medicine, Shenyang, Liaoning Province, China; b Affiliated Hospital of Liaoning University of Traditional Chinese Medicine, Shenyang, Liaoning Province, China.

**Keywords:** diabetic enteropathy, diarrhea, molecular docking, network pharmacology, Sishen pills

## Abstract

This study aimed to explore the effectiveness and safety of Sishen pills for the treatment of diarrheal diabetic enteropathy (DDE).

The Traditional Chinese Medicine (TCM) Systems Pharmacology and BATMAN-TCM databases were used to determine the chemical composition of Sishen pills and thus predict information on protein targets. We searched for potential targets of DDE in the GeneCards, DrugBank, Therapeutic Target (TTD), and DisGeNET databases. Using the intersection of the drug and disease targets, protein–protein interaction (PPI) networks and molecular interaction modules were constructed, and key targets were screened. The intersecting gene targets were imported into the Metascape database to conduct Gene Ontology (GO) and Kyoto Encyclopedia of Genes and Genomes (KEGG) pathway enrichment analyses. The core targets and active ingredients were then docked at the molecular level.

Sishen pills contain 70 active ingredients, 463 targets, and 566 disease targets. A module analysis of the targets revealed that the module was mainly related to adrenergic receptor activity, the adenosine phosphate kinase signaling pathway, and the G protein-coupled receptor signaling pathway. The GO and KEGG pathway enrichment results indicated that the protein genes regulated by Sishen pills were mainly enriched in the response to lipopolysaccharides, the AMPK signaling pathway, the JAK-STAT signaling pathway, and other signaling pathways. The molecular docking results showed that the core active compounds exhibited good binding activity with the predicted targets.

Sishen pills can regulate the immune function of the body through anti-inflammatory and antibacterial effects for the treatment of DDE.

## 1. Introduction

Diabetic enteropathy (DE), a chronic nonspecific inflammatory disease, is a common complication of diabetes mellitus. The main clinical manifestations are intractable painless diarrhea, watery loose stools, and alternating diarrhea and constipation, and a few patients present symptoms of anal incontinence.^[1]^ As the disease progresses, severe diarrhea and steatorrhea can occur. DE is more common in diabetic patients who are dependent on insulin, have a long course of diabetes, and have unstable blood sugar levels. Diabetic diarrhea has been experienced by approximately 10% to 20% of patients with diabetes.^[2]^ Despite advances in modern medicine, the pathogenesis of this disease is not fully understood. Many studies have shown that the pathogenesis of DE mainly includes hyperglycemia, gastrointestinal autonomic neuropathy, gastrointestinal microangiopathy, gastrointestinal smooth muscle changes, imbalance of intestinal flora, mental factors, and inflammation.^[3,4]^ Routine stool examination yields mostly normal results. A barium meal examination of the digestive tract may show signs of small intestinal malabsorption, and colonoscopy may identify signs of colonic mucosal congestion and edema. This complication not only affects the quality of life of patients with diabetes but also hinders the effective control of diabetes. Modern medicine is mostly based on control of the primary disease, the use of nutrient vessels, improvements in the microcirculation, adjustment of the microecology, and other drug therapies. At present, no specific Western medicine drugs are available to treat this condition; however, the long-term use of medications can produce drug resistance and results in repeated effects, and thus, the disease easily relapses. The occurrence of long-term severe diarrheal diabetic enteropathy (DDE) may cause ion disorders, acid-base imbalance, dehydration, and even death, which seriously affects the quality of life of the patients and can be life threatening. Traditional Chinese medicine (TCM) has a clear clinical effect on the treatment of DDE and few side effects. Therefore, there is an urgent need to develop effective TCM for the treatment of DDE and to meet the actual clinical needs.

DDE belongs to the categmedicineory of “diarrhea” in Chinese medicine. TCM principles suggest that the onset of this disease is closely related to the spleen and kidney, and Sishen pills are a classic prescription for the treatment of diarrhea. This treatment is a combination of Ershen pills and Wu Wei Zi powder known as Puji’s Prescriptions. Later generations supplemented and improved these prescriptions with reference to “Zhengsheng” to obtain the current Sishen pills, which can relieve diarrhea while affecting astringent intestines. Warming the kidney and the spleen simultaneously yields a remarkable effect. Modern medicine uses Sishen pills for the treatment of intestinal diseases such as ulcerative colitis (UC),^[5]^ allergic colitis,^[6]^ diarrheal irritable bowel syndrome,^[7]^ and postoperative diarrhea after colorectal cancer.^[8]^ Modern pharmacology shows that Sishen pills can inhibit excessive activation of inflammatory pathways,^[9–11]^ prevent cancer to a certain extent, regulate the body’s immune balance, repair the intestinal mucosal barrier, inhibit intestinal propulsion, improve gastrointestinal function, and effectively treat intestinal inflammatory diseases. Among the components, single herbs exhibit different degrees of antidiarrheal, anti-inflammatory, immune regulation, and other effects.

Compared with the single-target therapeutic effect of chemical drugs, TCM compound prescriptions are generally composed of several TCMs and include more components, complex structures, and different properties. Compound treatments exert multicomponent, multitarget, and multichannel overall therapeutic effects in the treatment of complex chronic diseases with multiple causes. This study aimed to use a combination of network pharmacology and molecular docking methods to gain an in-depth understanding of the mechanism of action of Sishen pills in the treatment of DDE and to provide a reliable basis for further clinical research.

## 2. Materials and Methods

### 2.1 Active ingredients in Sishen pills and target screening

The TCM system pharmacology database platform (TCMSP, http://tcmspw.com/tcmsp.php) and TCM molecular mechanism biological information analysis tool (BATMAN-TCM, http://bionet.ncpsb.org/batman-tcm/) were used to search for the chemical components and protein targets of each Chinese medicinal component of Sishen pills (composed of psoralen, Evodia, nutmeg, schisandra, ginger, and jujube). By analyzing the results from previous studies on the pharmacokinetics (absorption, distribution, metabolism and excretion; ADME) of the active ingredients of TCMs,^[12]^ the TCMSP database sets the oral bioavailability (OB) of drugs to ≥30% and the drug-likeness (DL) of biologically active molecules to ≥0.18. The TCMSP database was used to screen the active ingredients of TCMs and obtain the corresponding protein target information. Additionally, the BATMAN database uses a score cutoff ≥20 and a *P* value cutoff <.05 as restrictive conditions for screening active ingredients and target proteins.^[13]^ The literature was consulted to complement unpredicted chemical components with a certain potential value. After screening, to standardize the protein target information, the UniProt database (https://www.UniProt.org) was utilized to standardize the protein targets of the chemical components of the drug.

### 2.2 Screening of targets for DDE

Using “diarrheal diabetic enteropathy” as a keyword, the GeneCards database (https://www.genecards.org), DrugBank database (https://www.drugbank.ca), Therapeutic Target Database (http://bidd.nus.edu.sg/group/cjttd) and DisGeNET database (https://www.disgenet.org/) were utilized to identify potential targets for DDE treatment. In the GeneCards database, the score value is directly proportional to the relationship between the target and the disease. A higher score indicates a closer connection. Based on experience, a score ≥13.405 was considered to indicate a potential target of DDE.

### 2.3 Construction of a protein–protein interaction (PPI) network of the targets of the active ingredients of Sishen pills for DDE

The online software Venny 2.1.0 (https://bioinfogp.cnb.csic.es/tools/venny/) was used to intersect the DDE and TCM target genes with the aim of obtaining the common target genes of DDE and Sishen pills, that is, the target genes of Sishen pills in treating DDE. R language was used to draw a Venn diagram of the common targets. A total of 120 common targets were identified. These targets were imported into the STRING11.0 database (https://string-db.org). The PPI relationships were obtained in TSV format, and Cytoscape 3.8.0 software was used to construct the PPI network diagram. The MCODE plug-in based on the K-core algorithm was used to construct the molecular interaction module in the PPI network. Potential protein functional modules were obtained, and their molecular functions were described. Network analysis and the CytoNAC plug-in were used to analyze the topological parameters related to the target.

### 2.4 Pathway enrichment analysis

To explore the roles of the proteins present in Sishen pills in the treatment of DDE based on gene function, selected targets were imported into the Metascape database (http://metascape.org/gp/index.html). Enrichment analyses of the target genes were performed using the Kyoto Encyclopedia of Genes and Genomes (KEGG) and Gene Ontology (GO), and R language was used to transform the results of the enrichment analysis into a bubble chart.

### 2.5 Construction of the Sishen pill active ingredient-target network diagram

The Sishen pill active ingredient-target network diagram was constructed using Cytoscape 3.7.1 software, and a built-in software plug-in was used to analyze the network topology and the significance of the active ingredients and drug targets according to the network topology parameters.

### 2.6 Composition-target molecular docking

The Protein Data Bank (PDB) format of the 3D structure of each target gene was downloaded from the PDB database (https://www.rcsb.org/). The data were saved as a PDB format file and used to perform operations such as water removal and hydrogenation of the proteins.^[14]^ The SDF format file that screened out the 3D structures of key compounds was downloaded from the PubChem database (https://pubchem.ncbi.nlm.nih.gov/). The small-molecule SDF file was converted into a mol2 format file using open Babel software. AutoDock software was used to process the compounds and target proteins, the data were converted to pdbqt format, and Vina was utilized for docking. A binding energy lower than zero indicated that the ligand and receptor could bind spontaneously. Currently, there is no unified standard for the targeted screening of active molecules. According to the literature, active ingredients with a binding energy ≤–5.0 kJ/mol were selected as the screening basis for Sishen pills in the treatment of DDE.^[15]^

## 3. Results

After screening using the TCMSP and BATMAN-TCM databases, duplicate values were merged and deleted to obtain a total of 70 active ingredients in Sishen pills, and these ingredients included 14 compounds related to psoralen, 8 compounds related to nutmeg, 24 compounds related to Evodia, 8 compounds related to Schisandra, 4 compounds related to ginger, and 19 compounds related to dates (Table 1). The protein targets were matched using the UniProt database. After removing duplicate values and invalid targets, 463 targets were obtained. Psoralen had 274 targets, nutmeg had 66 targets, Evodia edulis had 201 targets, Schisandra had 20 targets, ginger had 54 targets, and jujube had 204 targets.

**Table 1:** Chinese medicine powder.

Mark	Active ingredients of psoralen		Database
BGZ13	Corylifolinin		BATMAN-TCM
BGZ1	Sophoracoumestan A		BATMAN-TCM
BGZ2	Isopsoralidin		BATMAN-TCM
BGZ3	Bavachin		BATMAN-TCM
BGZ4	Bakuchiol		BATMAN-TCM
BGZ5	Isobavachin		BATMAN-TCM
BGZ6	Bavachalcone		BATMAN-TCM
BGZ7	Bavachromene		BATMAN-TCM
BGZ8	Psoralidin		BATMAN-TCM
B	Stigmasterol		BATMAN-TCM
BGZ9	Xanthotoxin		BATMAN-TCM
BGZ10	Backuchiol		BATMAN-TCM
BGZ11	Angelicin		BATMAN-TCM
BGZ12	Isobavachalcone		BATMAN-TCM

Nutmeg active ingredients			
C		β-Sitosterol	TCMSP
RDK1		Isoguaiacin	TCMSP
RDK2		Galbacin	TCMSP
RDK3		5-[(2*S*,3*S*)-7-methoxy-3-methyl-5-[(*E*)-prop-1-enyl]-2,3-dihydrobenzofuran-2-yl]-1,3-benzodioxole	TCMSP
RDK4		Kudos	TCMSP
RDK5		Saucernetindiol	TCMSP
RDK6		Tetrahydrofuroguaiacin B	TCMSP
RDK7		Threo-austrobailignan-5	TCMSP

Evodia active ingredients			
A1		Berberine	TCMSP
WZY1		Rutaecarpine	TCMSP
WZY2		Isorhamnetin	TCMSP
C		β-Sitosterol	TCMSP
WZY3		Sitosterol	TCMSP
WZY4		Rutalinidine	TCMSP
WZY5		1-Methyl-2-[(*Z*)-pentadec-10-enyl]-4-quinolone	TCMSP
WZY6		Dihydrorutaecarpine	TCMSP
WZY7		1-Methyl-2-pentadecyl-4-quinolone	TCMSP
WZY8		Evodiamine	TCMSP
WZY9		1-(5,7,8-Trimethoxy-2,2-dimethylchromen-6-yl)ethanone	TCMSP
WZY10		Hydroxyevodiamine	TCMSP
WZY11		1-Methyl-2-undecyl-4-quinolone	TCMSP
WZY12		1-Methyl-2-nonyl-4-quinolone	TCMSP
WZY13		Evocarpine	TCMSP
WZY14		24-Methyl-31-norlanost-9(11)-enol	TCMSP
WZY15		6-OH-Luteolin	TCMSP
WZY16		Evodiamide	TCMSP
WZY17		Goshuyuamide I	TCMSP
WZY18		GoshuyuamideII	TCMSP
WZY19		Gossypetin	TCMSP
WZY20		Gravacridoneshlirine	TCMSP
WZY21		*N*-(2-Methylaminobenzoyl)tryptamine	TCMSP
A2		Quercetin	TCMSP

Schisandra active ingredients			
WWZ1		Longikaurin A	TCMSP
WWZ2		Deoxyharringtonine	TCMSP
WWZ3		Angeloylgomisin O	TCMSP
WWZ4		Schizandrer B	TCMSP
WWZ5		Gomisin-A	TCMSP
WWZ6		Gomisin G	TCMSP
WWZ7		Gomisin R	TCMSP
WWZ8		Wuweizisu C	TCMSP

Ginger active ingredients			
C		β-Sitosterol	TCMSP
SJ1		6-Methylgingediacetate2	TCMSP
B		Stigmasterol	TCMSP
SJ2		Poriferast-5-en-3 β-ol	TCMSP

Jujube active ingredients			
DZ1		Stepharine	TCMSP
DZ2		Zizyphus saponin I_qt	TCMSP
DZ3		Coumestrol	TCMSP
DZ4		Daechuine S7	TCMSP
DZ5		Jujubasaponin V_qt	TCMSP
DZ6		Mauritine D	TCMSP
A1		Berberine	TCMSP
DZ7		(*S*) -Coclaurine	TCMSP
DZ8		Mairin	TCMSP
B		Stigmasterol	TCMSP
C		β-Sitosterol	TCMSP
DZ9		Ruvoside_qt	TCMSP
DZ10		(+)-Catechin	TCMSP
DZ11		Stepholidine	TCMSP
DZ12		Nuciferin	TCMSP
DZ13		Fumarine	TCMSP
DZ14		β-Carotene	TCMSP
DZ15		(–)-Catechin	TCMSP
A2		Quercetin	TCMSP

We searched for disease targets of DDE in the disease target database and retrieved 4429 targets from GeneCards, 16 targets from DrugBank, no targets from TTD, and 1 target from DisGeNET. After screening and removing duplicate values, 566 disease targets were obtained, and these included 554 targets from GeneCards and 16 targets from DrugBank.

**Figure 1.**
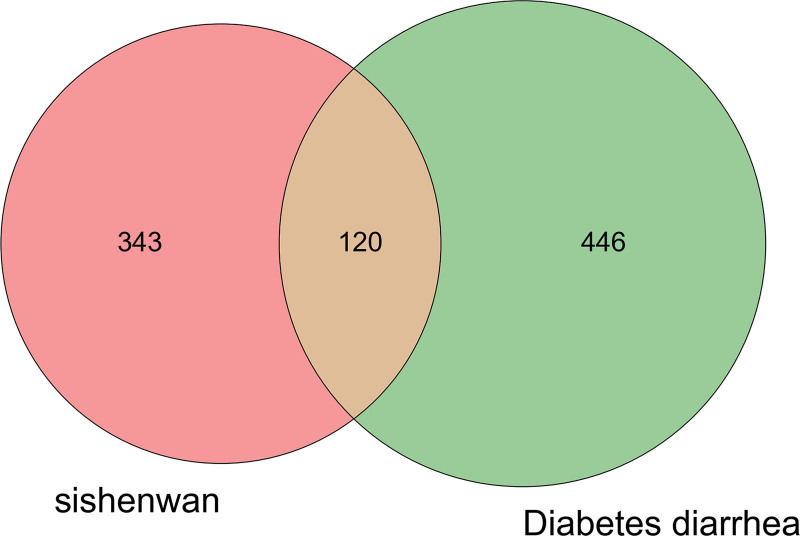
Venn diagram.

**Figure 2.**
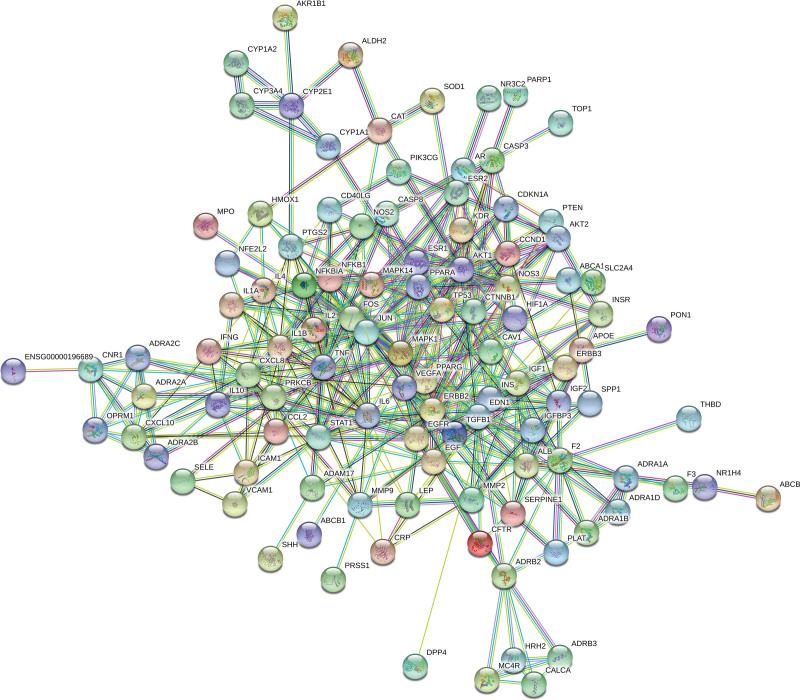
PPI network diagram of the cotarget genes. PPI = protein–protein interaction.

**Figure 3.**
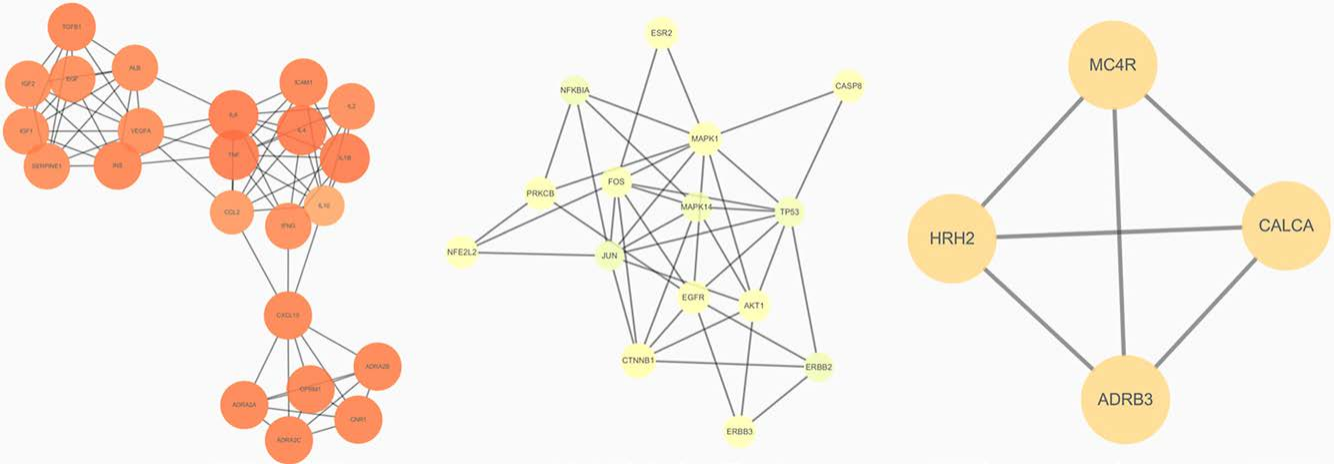
Protein function modules.

The Venn diagram identified 120 cotarget genes for Sishen pills and DDE (Fig. 1), which are potential targets for Sishen pills in the treatment of DDE. These targets were imported into the STRING11.0 database to obtain a network diagram (Fig. 2). The figure includes 76 targets where the proteins interact. The 166 edges represent the interaction relationships between the proteins. The average degree of the targets was 4.37, and the average clustering coefficient was 0.505. A higher network topology parameter indicates a stronger connection between the 2 targets and a closer interaction. The Cytoscape 3.7.1 MCODE plug-in was used to select the functional groups, retain the 3 best-rated modules, and describe the functions (Fig. 3 and Table 2). After module analysis of the targets, the 3 modules were primarily enriched. Module 1 was mainly related to adrenergic receptor activity. Module 2 was the adenosine phosphate kinase signaling pathway. Module 3 was a G protein-coupled receptor signaling pathway.

**Tables 2:** Molecular function description table of the protein modules.

Molecular function
Alpha2-adrenergic receptor activity
MAPK activity
G protein-coupled receptor binding

MAPK = mitogen-activated protein.

Using the Cytoscape tool to calculate the attribute value of each node, information was obtained for 19 main targets: TNF, AKT1, JUN, IL-6, VEGFA, TP53, MAPK1, INS, CXCL8, EGFR, MAPK14, EGF, EDN1, FOS, CTNNB1, IGF1, IL1B, F2, and ALB (Table 3). It is speculated that these targets may be key targets for the treatment of DDE using Sishen pills.

**Table 3:** Key target information.

Name	Degree	Betweenness	Closeness
TNF	30	1061.22518	0.493023256
AKT1	30	1317.280333	0.486238532
JUN	29	831.6593689	0.509615385
IL-6	27	1009.95606	0.493023256
VEGFA	23	724.1759745	0.5
TP53	21	671.3859273	0.466960352
MAPK1	21	724.6618487	0.486238532
INS	20	572.9208202	0.460869565
CXCL8	20	995.2608107	0.447257384
EGFR	20	619.0523884	0.471111111
MAPK14	20	533.7856554	0.471111111
EGF	19	642.3217511	0.469026549
EDN1	17	878.0029932	0.460869565
FOS	17	211.3487166	0.449152542
CTNNB1	16	214.0368913	0.439834025
IGF1	16	225.5731944	0.449152542
IL1B	16	102.5362619	0.429149798
F2	15	846.4041703	0.410852713
ALB	14	610.6252906	0.429149798

**Figure 4.**
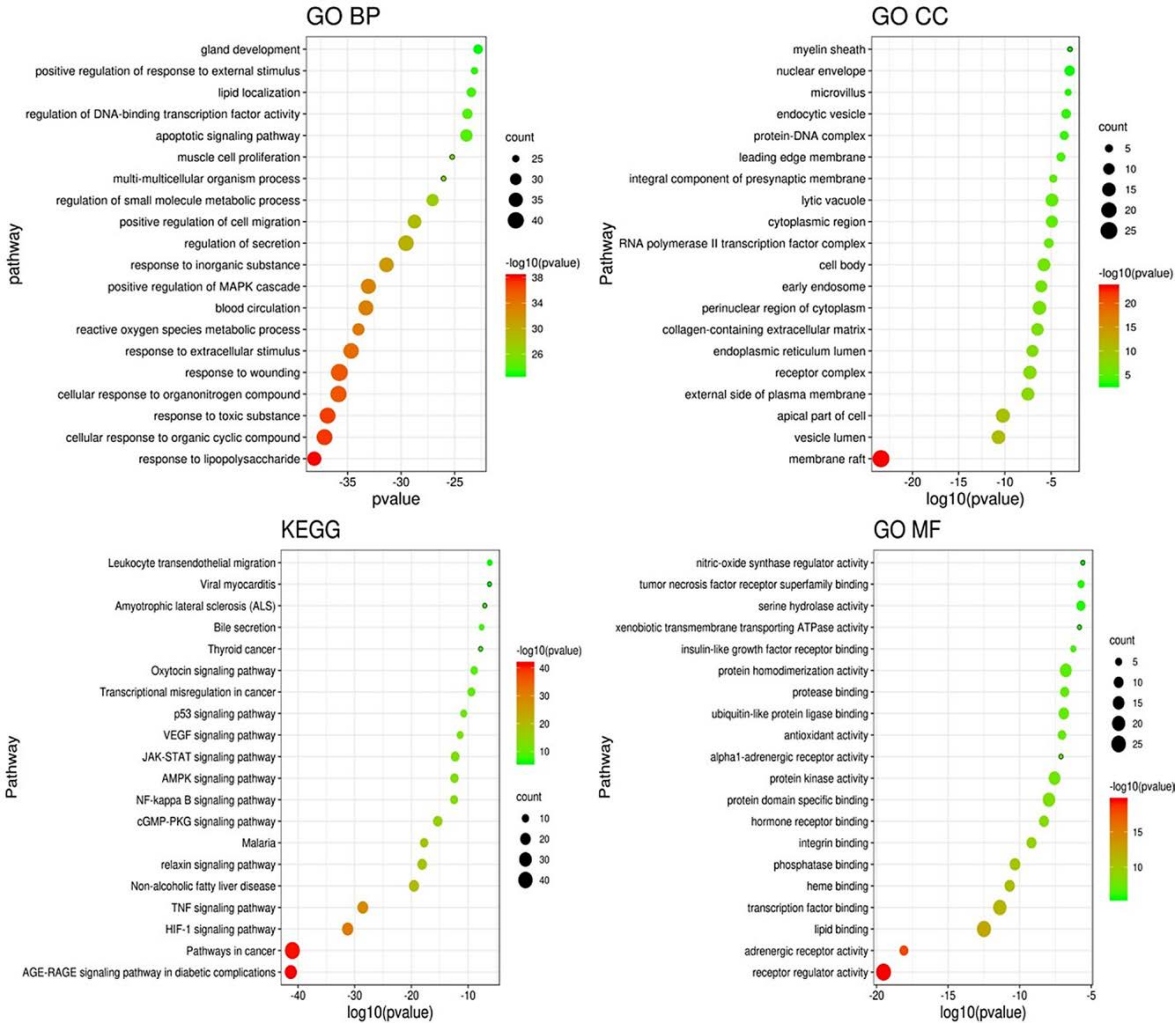
GO and KEGG enrichment analyses. GO = gene ontology, KEGG = Kyoto Encyclopedia of Genes and Genomes.

GO functional annotation analysis and KEGG pathway enrichment analysis using the Metascape platform was performed with the relevant targets of Sishen pills in the treatment of DDE. The top 20 GO analyses and enrichment pathways were visualized, and a column bubble chart of the GO and KEGG enrichment analyses was obtained (Fig. 4). The abscissa represents the size of the *P* value, the ordinate represents the channel name, and the size of the bubble graph represents the number of counts. A darker color indicates a smaller *P* value and thus a higher degree of enrichment. An increased pathway enrichment indicates that Sishen pills are more likely to play a role in treating diseases through these pathways.

**Figure 5.**
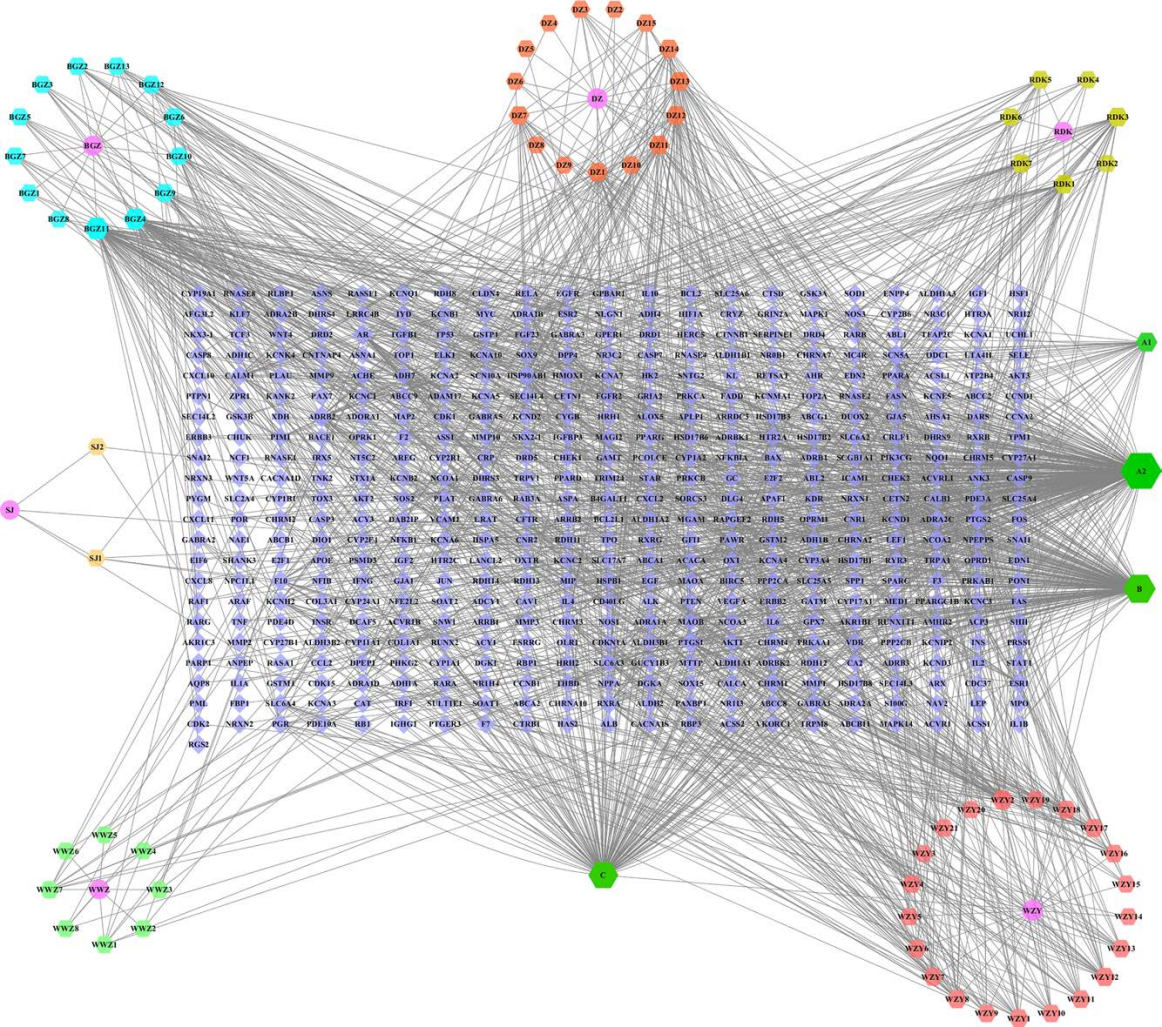
Active ingredient-target network diagram of Sishen pills.

The Sishen pill active ingredient-target network diagram has 539 nodes and 1594 edges, including 70 active ingredients and 463 gene targets (see Fig. 5). The edges represent the interactions between nodes, the diamonds represent genes, the hexagons represent active ingredients in TCM, and the circles represent TCM. The degree of the node in the graph was calculated. A higher degree value indicates that the node plays a greater role in the treatment of DDEs. The sizes were arranged in an orderly manner according to their degree values. A larger degree value indicates a larger shape. The top 3 active ingredients and targets, in order of degree value, were quercetin, stigmasterol, and β-sitosterol and PTGS2, SCN5A, and ADRB2, respectively.

**Figure 6.**
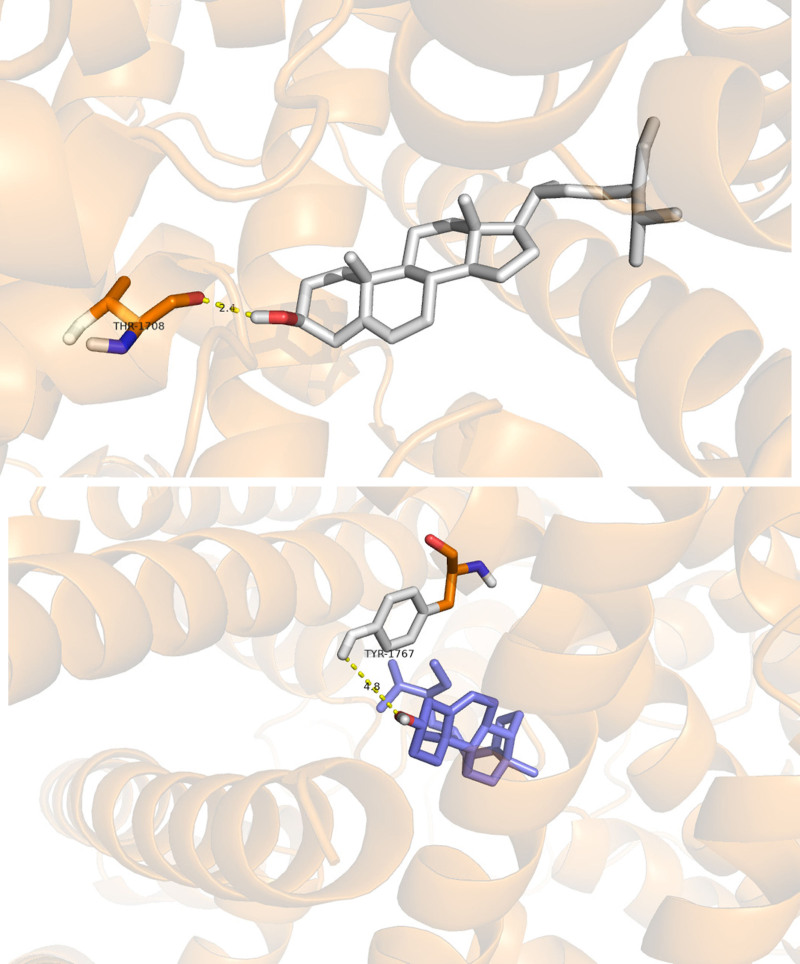
(A) Stigmasterol-SCN5A. (B) β-Sitosterol-SCN5A.

The top 3 target genes and components in the network graph were selected for molecular docking analysis. Three active ingredients, namely, quercetin, stigmasterol, and β-sitosterol, were used as candidate docking ingredients. PTGS2, SCN5A, and ADRB2 were selected as candidate targets. The molecular docking results showed that all the active ingredients with the exception of β-sitosterol and PTGS2 exhibited strong binding abilities to the targets, as shown in Table 4. PyMOL software was used to visualize the docking results of the components with the strongest binding energies and targets, as shown in Figure 6. These data indicated that the core active compound of Sishen pills exhibited good binding activity with the predicted target and thus suggest that Sishen pills may be a potential drug for the treatment of DDE.

**Table 4:** Molecular docking energy.

Binding energy (kJ mol–^1^)	Quercetin	Stigmasterol	β -Sitosterol
PTGS2	–7.03	–9.62	4.6
SCN5A	–6.65	–39.75	–35.56
ADRB2	–8.58	–24.27	–15.9

## 4. Discussion

### 4.1. Analysis of the composition of Sishen pills

The main components of psoralen are flavonoids, coumarins, and monoterpene phenols, which suppress cancer, enhance immunity, and exert anti-inflammatory, antibacterial, antioxidant, antidepressant, neuroprotective, and liver function effects.^[16]^ Studies have shown that the chemical components of different psoralen compounds, such as isopsoralen, psoralidin, isobaravachin, and corylifolinin, exhibit good antioxidant effects.^[17]^ Among these components, psoralen exerts the strongest antioxidant effect, can improve glucose tolerance through antioxidant stress and can delay the progression of diabetes complications. Psoralen and isopsoralen in psoralen extract can inhibit H_2_O_2_-induced islet B-cell death by reducing the level of reactive oxygen species (ROS) and activating antioxidant enzymes and by significantly improving the blood glucose tolerance and serum insulin levels in diabetic mice.^[18]^ The coumarin ingredients include psoralidin, isopsoralidin, and sophoracoumestan A, which exert a significant inhibitory effect on gram-positive and gram-negative bacteria^[19]^ and thereby affect the intestinal flora. Psoralen, isopsoralen, and bakuchiol have regulatory effects on a variety of immune cells and can inhibit the secretion of macrophage inflammatory factors, including TNF-α, IL-1β, and IL-6.^[20]^ Nutmeg has antibacterial, anti-inflammatory, antioxidant, anticancer, antidiarrheal, antiulcer, and other biological activities.^[21]^ The nutmeg stock solution and the petroleum ether extract in nutmeg can reduce the average number of loose stools in guinea pigs and increase the incubation period, and the stock solution also exerts a significant analgesic effect.^[22]^ Modern pharmacological studies have shown that the alkaloids in Evodia are the main active ingredients and are present at the highest concentrations. The active ingredients of Evodia exert anti-inflammatory, antibacterial, antiulcer, analgesic, and 2-way gastrointestinal regulatory effects.^[23]^ Evodia has a wide range of functions in the digestive system and can regulate the function of the gastrointestinal tract in both stimulatory and inhibitory directions. Evodia at low concentrations promotes intestinal peristalsis, whereas high concentrations inhibit intestinal peristalsis.^[24,25]^ Modern pharmacology has confirmed that anti-inflammatory drugs, such as steroids, nonsteroids, and berberine, can be used to treat diarrhea. Therefore, the therapeutic effect of Evodia may be achieved by regulating gastrointestinal function through its anti-inflammatory effects. Schizandrin B significantly scavenges free radicals, inhibits lipid peroxidation, improves the antioxidant capacity of rat brain cell mitochondria, and exerts antioxidant effects through antilipid peroxidation.^[26]^ Schisandra volatile oil lowers blood sugar. Schisandra acid polysaccharides can improve insulin resistance and lower blood sugar levels in type 2 diabetic rats by inhibiting inflammation.^[27]^ The above results suggest that the pharmacological basis of the prescription is its antibacterial and anti-inflammatory effects, which can improve digestive tract symptoms, regulate energy metabolism and regulate the immune function of the body.

### 4.2. Analysis of key targets and pathways

The occurrence and development of DDE are related to intestinal microbes. The microbiota is essential not only for maintaining the stability of the intestinal environment but also for regulating the immune system and inflammatory factors. Abnormal intestinal microbes can lead to the occurrence of “metabolic endotoxemia,” which puts the body in a state of chronic low-grade inflammation. Endotoxins, also known as lipopolysaccharides (LPSs), are the main component of the cell wall of gram-negative bacteria. LPS is released into the blood when the bacteria die and dissolve. After LPS enters the blood and binds to LPS-binding protein, LPS is transported to interact with Toll-like receptor 4 (TLR-4) and CD14 on the surface of immune cells. TLR-4, as an earlier discovered TLR, activates nuclear factor-κB (NF-κB) by recognizing LPS, which results in activation of the expression of inflammatory cytokines and induction of an inflammatory response. Studies have shown that Sishen pills downregulate the expression of the linker protein TLR interacting protein, prevent binding to TLR-4, reduce the transcriptional activity of NF-κB, and inhibit activation of the NF-κB signaling pathway, which results in the interruption of downstream signal transduction, the inhibition of cell activation and the inflammatory response, and the promotion of colonic mucosal repair.^[28]^ The NF-κB pathway promotes the migration and aggregation of T lymphocytes,^[29]^ which causes these cells to secrete more cytokines and chemokines and forms a vicious cycle that leads to uncontrolled inflammation and an immune response.^[30]^ Related studies have found that Sishen pills can significantly reduce the serum IL-8 and TNF-α levels in enteritis model rats, downregulate the expression of NF-κB p65 protein in colon tissue, and improve colonic mucosal damage.^[31]^ Studies have found that the treatment of chronic refractory diarrhea with Sishen pills increases the abundances of symbiotic flora, such as Bifidobacterium and Lactobacillus, in the intestine to varying degrees, and decreases the abundances of pathogenic bacteria, such as *Escherichia coli*, to varying degrees. This finding shows that Sishen pills can regulate the intestinal flora, rebuild the microecological balance of the host, and fundamentally treat chronic refractory diarrhea.^[32]^

Adrenergic receptor activity is closely related to this disease. Under normal circumstances, the stimulation of α2-adrenergic receptors in intestinal cells can promote the absorption of intestinal juice. Studies have shown that adrenergic fibers that innervate intestinal cells are absent in patients with diabetes,^[33]^ which leads to obvious obstacles to intestinal fluid absorption in the ileum and colon and promotion of the occurrence of diarrhea. Chang et al^[34]^ proposed that DDE is caused by a weakened ability of adrenergic neurons to regulate mucosal ion transport and high postsynaptic denervation sensitivity. Clonidine hydrochloride can increase the number of α2-adrenergic receptors and reverse these changes. Altan et al^[35]^ found that diabetic gastrointestinal complications are related to decreased β-adrenergic and/or 5-H receptor activity. A reduction in receptor activity reduces the tension in the small intestine and accelerates its transport, resulting in faster intestinal peristalsis, which manifests as diarrhea and other symptoms.

PAR is a G protein-coupled receptor involved in the functional regulation of gastrointestinal sensation, intestinal mucosal inflammation response, and gastrointestinal smooth muscle movement and plays a regulatory role in functional gastrointestinal disease.^[36]^ PAR2 is widely distributed in the digestive tract, where it activates the PAR2 receptor of intestinal epithelial cells and stimulates the intestinal mucosa to secrete a large amount of water, which causes diarrhea.^[37]^ PAR2 can also be involved in the regulation of intestinal function by influencing intestinal motility and increasing intestinal mucosal permeability and exhibits high sensitivity.^[38]^ PAR4 mediates the synthesis and secretion of inflammatory mediators and cytokines in the intestine and thereby participates in the process of intestinal damage.^[39]^ PAR4 is activated and secretes acetylcholine and tachykinin, which contract the intestinal muscles, damage the intestine, and cause an inflammatory response to cause intestinal dysmotility.^[39]^ Sishen pills can reduce the levels of PAR2 and PAR4 in the rectal mucosa of patients, inhibit the inflammatory response, regulate intestinal function, and treat intestinal diseases.^[40]^

Protein kinase G (PKG) and its signaling pathway, nitric oxide (NO)-sGC-c GMP-PKG, rely on the normal expression of NO. NO activates soluble guanylate cyclase (sGC), which catalyzes intracellular cGMP synthesis. cGMP then crossacts with cAMP by regulating the activation of ion channels and/or the activity of cGMP-dependent protein kinase (PKG), which is important for maintenance of the normal physiological movement of the gastrointestinal tract.^[30]^ Among these compounds, NO, as an inflammatory transmitter and immune molecule, can affect intracellular DNA replication and cellular energy metabolism, which can reduce the ability of antioxidant substances such as SOD to eliminate oxygen free radicals and induce cytotoxicity. Studies have found that Sishen pills can reduce the concentration and activity of NO, decrease the NO levels, enhance SOD vitality, stop lipid peroxidation, reduce cytotoxicity, inhibit inflammation, and repair the intestinal mucosa.^[41]^

Immune dysfunction of the intestinal mucosa can directly or indirectly affect intestinal motility and barrier function in patients with DDE. Studies have found that immune dysfunction of the intestinal mucosa is related to an excessive release of inflammatory cytokines. In addition, high expression of TNF-α, IL-6, IL-8, and other inflammatory factors plays an important role in the occurrence and development of inflammatory bowel disease.^[42]^ These factors affect intestinal motility through nerve and endocrine pathways to act on the nerve fibers of the intestinal mucosal and smooth muscle layers, which results in symptoms such as abdominal pain, bloating, and changes in stool characteristics. An imbalance between proinflammatory and anti-inflammatory factors is important in the pathogenesis of this disease. The presence of proinflammatory cytokines produced by monocytes and macrophages, such as IL-1, IL-6, IL-8, IL-17, and TNF-α, in the serum can mediate the occurrence of this disease. Anti-inflammatory cytokines, such as IL-4, IL-10, IL-13, and transforming growth factor-β, can maintain intestinal immune function and homeostasis and are mainly produced by T cells.^[43]^ TNF-α is an important proinflammatory factor. TNF-α can enhance the phagocytic function of mononuclear macrophages, stimulate the synthesis and release of IL-8, IL-1 and other cytokines, produce a cascading effect of cytokines, and promote expansion of the inflammatory response;^[44]^ these effects induce occurrence of intestinal cell damage and colonic epithelial cell apoptosis. Papadakis and Targan^[45]^ found that the serum TNF-α levels in patients with inflammatory bowel disease are elevated. High concentrations of TNF-α can stimulate epithelial cells on the surface of the intestinal mucosa, and under the combined action of other mechanisms, increased intestinal mucosal immune dysfunction can induce inflammation of the intestinal mucosa, which can easily lead to the aggravation of clinical symptoms. IL-8 induces inflammation by mediating low concentrations of TNF-α, which is closely associated with the pathogenesis of intestinal inflammation. Imada et al^[46]^ found that the expression of IL-8 mRNA in patients with UC is significantly higher than that in the control group. Gao et al^[31]^ observed that the levels of serum IL-8 and TNF-α in rats with inflammatory bowel disease are significantly higher than those in the blank group. Treatment with Sishen pills significantly reduced the levels of serum IL-8 and TNF-α in rats. This result indicates that the mechanism of action of Sishenwan in the treatment of intestinal inflammation is related to reductions in the IL-8 and TNF-α levels. In addition, Sishen pills can inhibit the expression of the proinflammatory factors IL-1β and TNF-α and increase the expression of the anti-inflammatory factors IL-4 and IL-13, which adjusts the balance between proinflammatory and anti-inflammatory factors.

The PI3K/Akt/mTOR signaling pathway affects cell proliferation, differentiation, and apoptosis and plays an important role in intestinal inflammation and the tumor response.^[47]^ Studies have found that inhibition of the PI3K/Akt/mTOR signaling pathway can also induce autophagy in macrophages to inhibit inflammation.^[48]^ Related studies have found that the anti-inflammatory cytokine IL-10 can inhibit autophagy induced by interferon (IFN)-γ by activating the PI3K/Akt signaling pathway.^[49]^ Studies have shown that activation of the PI3K/Akt pathway inhibits intestinal mucosal NF-kB p65 and thereby reduces the expression of TNF-α and IL-1β. This process can alleviate intestinal inflammation and reduce the disease activity index of diseased mice.^[50]^ Studies have shown that Sishen pills can effectively inhibit the activation of the PI3K/Akt/mTOR signaling pathway, regulate the proinflammatory cytokine IL-1β and the anti-inflammatory cytokine IL-10, alleviate inflammation, regulate immunity, and promote intestinal mucosal damage repair, which results in improvements in clinical symptoms.^[51]^

The JAK/STAT signaling pathway is involved in a variety of physiological and pathological processes, such as cell proliferation, differentiation, apoptosis, immune regulation, inflammation, and tumors.^[52]^ The JAK/STAT signaling pathway plays an important role in inhibiting intestinal mucosal inflammation and maintaining the intestinal epithelial immune balance. Under normal circumstances, activation of the JAK/STAT signaling pathway reduces the inflammatory response and promotes the recovery of intestinal inflammation. However, excessive activation of the JAK/STAT signaling pathway disrupts this balance. The intestine loses the ability to tolerate normal flora, which leads to more severe inflammation.^[53]^ The mucosal cells of the intestine of patients with inflammatory bowel disease exhibit upregulated expression of STAT1. STAT1 inhibits the signal transduction process of IFN, upregulates the expression of caspase 1 and Fas/FasL, inhibits growth, stimulates apoptosis, and promotes intestinal ulcers.^[54]^ The activation of STAT3 in the mucosal cells of the intestine of patients can upregulate Bcl-2 expression, downregulate Bax expression, resist apoptosis, and promote the proliferation of cells. Therefore, the JAK/STAT signaling pathway should be accurately negatively regulated and should be terminated in time to prevent an excessive immune response.^[55]^ Suppressor of cytokine signaling (SOCS) is a negative regulator that acts directly on the JAK/STAT signaling pathway.^[56]^ By participating in the negative regulation of a variety of cytokine-mediated pathways, SOCS ultimately affects cell proliferation, differentiation and apoptosis. The results showed that Sishen pills can upregulate the contents of SOCS mRNA and SOCS2/3 protein in the colon tissue of UC rats,^[57]^ negatively regulate the JAK/STAT signaling pathway, and inhibit the development of inflammation, which results in restoration of the balance of intestinal mucosal immune homeostasis.

Apoptosis is an important mechanism that causes intestinal mucosal damage and immune disorders in intestinal inflammatory diseases.^[58]^ FAS is a membrane receptor protein that belongs to the NGF receptor superfamily, plays a signal transduction role in apoptosis and induces apoptosis in cells expressing the Fas protein.^[59]^ The Bcl-2 protein is a recognized apoptosis suppressor, and the Bcl-2/Bax ratio regulates the rate of apoptosis.^[60]^ Sensitized T cells can induce colonic epithelial cell and protective Th2 cell apoptosis or even kill directly through the Fas/FasL pathway. Eventually, the colonic mucosal barrier is destroyed, leading to ulcer formation.^[61]^ Some studies have demonstrated that reduced Bax expression and an increased Bcl-2/Bax ratio are beneficial to resisting the occurrence of cell apoptosis in chronic inflammatory bowel disease.^[62]^ Studies have found that enema administration of Sishen pills can significantly alleviate damage to the colon mucosa, reduce the expression of Fas in colon tissue, and increase the expression of FasL. The expression of Bcl-2 mRNA was significantly increased by the high dose of Sishenwan, and the Bcl-2/Bax ratio was significantly increased by all doses, which suggested that the trend of its final action may be to resist the occurrence or aggravation of apoptosis in chronic inflammatory bowel disease. Studies have shown that Sishen pills may delay colonic epithelial cell apoptosis or promote inflammatory cell apoptosis by regulating the expression of Fas/FasL and Bax/Bcl-2 mRNAs in colon tissue.^[63]^

The p38 mitogen-activated protein kinase (MAPK) signaling pathway can stimulate the release of inflammatory, growth, cell stress, and other factors.^[59]^ p38 MAPK is necessary for Bax activation and apoptosis in vitro.^[60]^ Bcl-2 is an inhibitor of apoptosis. Increased expression of Bcl-2 can combine with Bax to form a more stable heterodimer to inhibit cell apoptosis; therefore, the Bcl-2/Bax ratio can regulate cell apoptosis.^[61]^ Caspase-, Bcl-2-, and p53 proteinase family-related genes were activated after Fas and FasL were integrated in the initial phase, whereas the overexpression of caspases subsequently activated the MAPK signaling pathway to phosphorylate p38 and c-Jun and induced the expression of transcription factors (p53 and FasL) to induce apoptosis. Relevant studies have shown that Sishen pills inhibit the expression of p53, caspase-3, c-Fos, c-Jun, TNF-α, and Bax mRNA,^[62]^ similar to the results reported by Liu et al,^[63]^ improve colitis in mice and increase the level of Bcl-2 mRNA and the Bcl-2/Bax ratio. Sishenwan also inhibits the expression of Fas and thereby reduces apoptosis in mouse colonic epithelial cells. Moreover, Sishenwan increases the mRNA expression of IL-4 and IL-10 and thus inhibits the production of proinflammatory factors (TNF-α and IL-1).^[64]^ Therefore, Sishenwan can effectively inhibit the mRNA expression of apoptosis-related molecules in the p38 MAPK signaling pathway and thereby decreases the apoptosis of colonic mucosal epithelial cells in mice with intestinal inflammation.

As an energy sensor, AMPK kinase can sense energy changes and maintain the energy balance under metabolic stress at the cellular and physiological levels. The regulation of TOR signaling by interfering with AMPK-TSC signaling affects the process of cell anabolism, which plays an important role in regulating the balance of cell energy metabolism. Studies have found that Evodia can increase the level of ROS in HCT-116 cells, activate AMPK signaling, inhibit mTOR activity, and cause autophagy.^[65]^ Psoralen dihydroflavonoids can activate the Akt and AMPK pathways to promote glucose uptake through the translocation of glucose transporter 4.^[66]^ Nutmeg can also inhibit the autophagic activity of cells by increasing the level of Akt protein and activating mTOR signaling.^[67]^ Schizandrin A can protect against brain ischemia/reperfusion (I/R) damage by inhibiting inflammation and oxidative stress, and this effect is regulated by the AMPK/Nrf2 pathway.^[68]^ Studies have shown that the role of Sishen pills in the treatment of inflammatory bowel disease is to activate AmpK-TSC signaling and inhibit PI3K-Akt signaling, and there effects regulate mTOR protein expression and its downstream signaling pathway, improve the expression level of ATP synthase subunits, intervene in cell-related energy metabolism pathways, affect the differentiation of immune memory T cells and their CD4+ and CD8+ subtypes, and ultimately treat inflammatory bowel disease.^[69]^

## 5. Conclusion

Network pharmacology studies have found that Sishen pills can treat DDE through multiple pathways, targets, and other synergistic effects. Imbalances in the intestinal flora, excessive release of inflammatory factors, and immune dysfunction can lead to changes in the intestinal environment, which leads to the occurrence of DDE. Sishen pills function in the treatment of DDE by regulating the immune function of the body and exerting anti-inflammatory and antibacterial effects.

## Author contributions

**Conceptualization:** Yunxia Tao.

**Data curation:** Chongchai Li.

**Formal analysis:** Yunxia Tao, Chongchai Li.

**Methodology:** Yunxia Tao, Tianshu Gao, Jingjing Huo.

**Supervision:** Tianshu Gao, Jingjing Huo.

**Writing – original draft:** Yunxia Tao.

**Writing – review & editing:** All authors.
